# Development and validation of a model based on preoperative dual-layer detector spectral computed tomography 3D VOI-based quantitative parameters to predict high Ki-67 proliferation index in pancreatic ductal adenocarcinoma

**DOI:** 10.1186/s13244-024-01864-9

**Published:** 2024-12-05

**Authors:** Dan Zeng, Jiayan Zhang, Zuhua Song, Qian Li, Dan Zhang, Xiaojiao Li, Youjia Wen, Xiaofang Ren, Xinwei Wang, Xiaodi Zhang, Zhuoyue Tang

**Affiliations:** 1 Department of Radiology, Chongqing General Hospital, Chongqing University, Chongqing, China; 2 Department of Clinical and Technical Support, Philips Healthcare, Chengdu, China

**Keywords:** Dual-layer detector spectral computed tomography, Ki-67, Pancreatic ductal adenocarcinoma, Prognosis, Nomogram

## Abstract

**Objective:**

To develop and validate a model integrating dual-layer detector spectral computed tomography (DLCT) three-dimensional (3D) volume of interest (VOI)-based quantitative parameters and clinical features for predicting Ki-67 proliferation index (PI) in pancreatic ductal adenocarcinoma (PDAC).

**Materials and methods:**

A total of 162 patients with histopathologically confirmed PDAC who underwent DLCT examination were included and allocated to the training (114) and validation (48) sets. 3D VOI-iodine concentration (IC), 3D VOI-slope of the spectral attenuation curves, and 3D VOI-effective atomic number were obtained from the portal venous phase. The significant clinical features and DLCT quantitative parameters were identified through univariate analysis and multivariate logistic regression. The discrimination capability and clinical applicability of the clinical, DLCT, and DLCT-clinical models were quantified by the Receiver Operating Characteristic curve (ROC) and Decision Curve Analysis (DCA), respectively. The optimal model was then used to develop a nomogram, with the goodness-of-fit evaluated through the calibration curve.

**Results:**

The DLCT-clinical model demonstrated superior predictive capability and a satisfactory net benefit for Ki-67 PI in PDAC compared to the clinical and DLCT models. The DLCT-clinical model integrating 3D VOI-IC and CA125 showed area under the ROC curves of 0.939 (95% CI, 0.895–0.982) and 0.915 (95% CI, 0.834–0.996) in the training and validation sets, respectively. The nomogram derived from the DLCT-clinical model exhibited favorable calibration, as depicted by the calibration curve.

**Conclusions:**

The proposed model based on DLCT 3D VOI-IC and CA125 is a non-invasive and effective preoperative prediction tool demonstrating favorable predictive performance for Ki-67 PI in PDAC.

**Critical relevance statement:**

The dual-layer detector spectral computed tomography-clinical model could help predict high Ki-67 PI in pancreatic ductal adenocarcinoma patients, which may help clinicians provide appropriate and individualized treatments.

**Key Points:**

- Dual-layer detector spectral CT (DLCT) could predict Ki-67 in pancreatic ductal adenocarcinoma (PDAC).
- The DLCT-clinical model improved the differential diagnosis of Ki-67.
- The nomogram showed satisfactory calibration and net benefit for discriminating Ki-67.

**Graphical Abstract:**

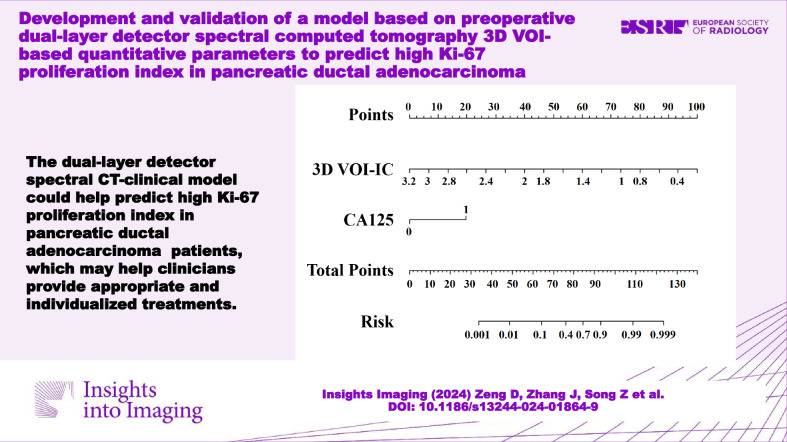

## Introduction

Pancreatic ductal adenocarcinoma (PDAC) is a highly fatal malignancy, being the fourth leading cause of cancer-related death globally [1]. It is notorious for its premature metastasis and latent progression, with a 5-year survival rate of merely 12% [2]. Ki-67 is a nuclear protein expressed throughout the cell cycle except in G0, enabling its use to distinguish between proliferating and quiescent cells [3]. Recent studies have shown a robust association of high Ki-67 proliferation index (PI) with poor prognosis in PDAC [4–6]. Considering the high risk of early postoperative recurrence in PDAC patients with a high Ki-67 PI [7], the preoperative identification of these individuals and the implementation of neoadjuvant therapy would likely yield superior clinical benefits compared to upfront surgery [8].

Immunohistochemical (IHC) examination is a common method for detecting Ki-67 [9]. However, it necessitates invasive procedures like surgery or percutaneous puncture biopsy to obtain samples. This carries the potential risk of complications, including surgical site infection and needle migration. Additionally, limitations such as some small sample sizes in biopsy (which may not accurately represent the entire tumor), tumor heterogeneity, and the operator’s technical proficiency and experience can affect diagnostic accuracy [10, 11]. Moreover, the time required to process and analyze biopsy samples may lead to delays in initiating appropriate treatment and further impact its effectiveness. Therefore, a non-invasive method for timely and accurate prediction of Ki-67 PI in PDAC is imperative. Currently, dual-layer detector spectral computed tomography (DLCT) enables multiparametric imaging to reflect tissue perfusion and can offer quantitative information about tissues. Some studies have shown that various quantitative parameters derived from DLCT have the potential to predict Ki-67 PI in a variety of malignant tumors [12–16]. However, in previous studies [12–16], parameters were often manually obtained from the region of interest (ROI) on the largest lesion slices, leading to potential measurement bias and overlooking information about the entire lesion’s tissue. Given the intralesional heterogeneity of tumors, analyzing the entire tumor in three dimensions is deemed superior to manual ROI analysis in two dimensions, which can provide more comprehensive information about the tumor [17]. Additionally, the three-dimensional (3D) analysis exhibits greater interobserver consistency while simultaneously demonstrating less variability and higher reproducibility in quantitative analysis [18]. However, it remains unclear whether DLCT 3D VOI-based quantitative parameters can accurately predict Ki-67 PI in PDAC.

In this study, we hypothesize that DLCT 3D VOI-based quantitative parameters can predict Ki-67 PI in PDAC. To test our hypothesis, we aimed to develop a model that combines DLCT 3D VOI-based quantitative parameters with clinical features to identify PDAC patients with a high Ki-67 PI and visualize it as a nomogram for helping clinicians optimize therapeutic strategies for PDAC.

## Materials and methods

### Patients

**Fig. 1:**
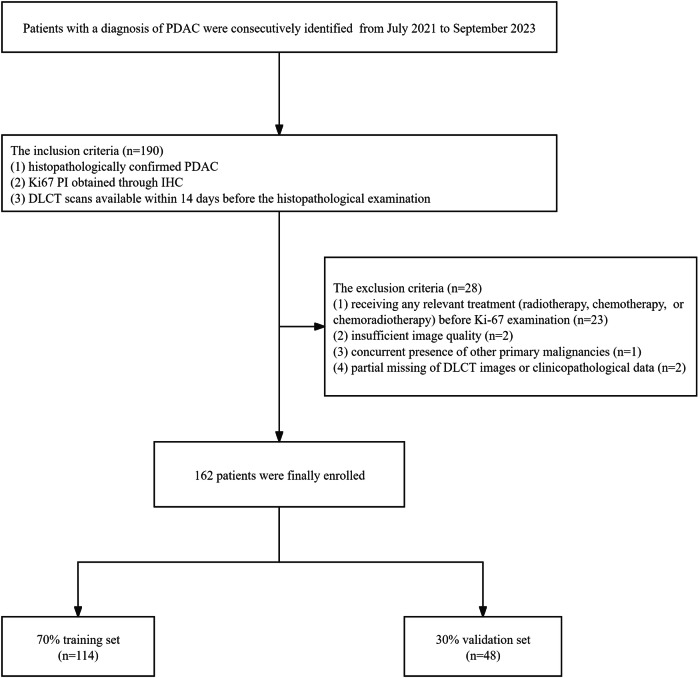
Flowchart for the study population. PDAC, pancreatic ductal adenocarcinoma; IHC, immunohistochemistry; DLCT, dual-layer detector spectral computed tomography; PI, proliferation index

The study obtained ethical approval from the institutional review board at Chongqing General Hospital and patient consent was waived for this retrospective study. Between July 2021 and September 2023 patients who were diagnosed with pathologically confirmed PDAC were enrolled. As depicted in Fig. 1, the inclusion criteria comprised: (1) histopathologically confirmed PDAC; (2) Ki-67 PI obtained through IHC; and (3) DLCT scans available within 14 days before the histopathological examination. The exclusion criteria included: (1) receiving any relevant treatment (radiotherapy, chemotherapy, or chemoradiotherapy) before Ki-67 examination; (2) insufficient image quality; (3) concurrent presence of other primary malignancies; and (4) partial missing of DLCT images or clinicopathological data. Eventually, a total of 162 patients (94 males and 68 females; mean age 62.08 ± 10.33 years) were included in the study and randomly allocated to a 7:3 ratio, with 114 in the training set and 48 in the validation set.

### Immunohistochemical analysis of Ki-67 PI

The Ki-67 in 162 patients were examined using IHC on surgical specimens (63.6%) and percutaneous or transgastric core biopsy specimens (36.4%). All specimens were derived from primary pancreatic tumors. Positive cells were identified as those with a brown nucleus. Ki-67 PI assessment involved calculating the percentage of positive cells among 1000 malignant cells (× 200). Given the absence of a predetermined optimal threshold, this study adopted a threshold of 50%, as suggested by previous research [8]. In our study, PDAC was categorized into either the low Ki-67 PI group (< 50%) or the high Ki-67 PI group (≥ 50%).

### DLCT image acquisition

All patients were scanned using DLCT (IQon spectral CT, Philips Healthcare, Amsterdam, The Netherlands). The scan range was from the top of the diaphragm to the inferior margin of the liver. The scanning protocol was as follows: tube voltage, 120 kV; tube current, smart mAs; rotation time, 0.5 s; detector collimation, 64 × 0.625 mm; field of view, 350 × 350 mm; matrix, 512 × 512; layer thickness, 5 mm. Patients were intravenously injected with non-ionic contrast media (Ioversol, 350 mgI/mL) at a dose of 1.5 mL/kg at an injection rate of 2.5–3.5 mL/s using the high-pressure automatic injector, then pumped 30 mL saline at the same rate. The arterial phase and portal vein phase (PVP) were performed 12 s and 35 seconds after the predetermined threshold of 150 HU within the aorta descendens (activated bolus tracking), respectively.

### Clinical features and quantitative imaging analysis

The clinical features including gender, age, body mass index, neutrophil-to-lymphocyte ratio, platelet-to-lymphocyte ratio, lymphocyte-monocyte ratio, platelet-to-white blood cell ratio, total bilirubin, Albumin, carbohydrate antigen (CA)19-9, CA125 and carcinoembryonic antigen (CEA) were retrieved from the hospital information system for each patient.

DLCT images were imported into the prototype software (IntelliSpace Discovery, Philips Healthcare, The Netherlands) and reconstructed into virtual monoenergetic images (40 keV and 100 keV), effective atomic number (Zeff) images, and iodine images for subsequent quantitative analysis. Two radiologists (R1 and R2, with 7 and over 11 years of experience in reading CT images, respectively) delineated the tumor contours in a slice-by-slice manner on the PVP images (Previous study has found PVP images may yield adequate tumor conspicuity, which may be conducive to delineate contours of tumor [19]), and then the software automatically generated DLCT 3D VOI-based quantitative parameters, including 3D VOI-HU40keV, 3D VOI-HU100keV, 3D VOI-Zeff, and 3D VOI-iodine concentration (IC). The 3D VOI-HU40keV and 3D VOI-HU100keV were chosen to calculate λHU, computed as follows: λHU = (3D VOI-HU40keV - 3D VOI-HU100keV) / (100–40). The intraclass correlation coefficient was used to analyze the consistency of the DLCT 3D VOI-based quantitative parameters extracted by the radiologists.

### Development and assessment of the models and nomogram

Univariate analysis was initially conducted to compare the differences in DLCT 3D VOI-based quantitative parameters and clinical features between the high and low Ki-67 PI groups. Subsequently, the identified significant variables were incorporated into regression analysis to determine independent predictors in the training set. The clinical model was then developed using these independent clinical features, and the DLCT model was developed based on independent DLCT 3D VOI-based quantitative parameters. Simultaneously, the DLCT-clinical model was developed by integrating all identified independent predictors. Receiver Operating Characteristic curve (ROC) and Decision Curve Analysis (DCA) were utilized to assess the discrimination capability and clinical applicability of the three models. The validation set independently verified each of the three models. The DeLong test was applied to compare the Area Under the Curve (AUC) of the three models. The optimal model was then utilized to develop a nomogram. Finally, the calibration curve was used to evaluate the calibration performance of the nomogram.

### Statistical analysis

All statistical analyses and calculations were performed using R software (http://www.R-project.org), SPSS software (version 26.0, SPSS, IBM), and MedCalc (version 18.2.1, MedCalc Software). The Shapiro-Wilk test was used to test the normality of the data. Normally distributed data were presented as mean ± standard deviation, while non-normally distributed data were expressed as median (25th, 75th percentiles). Categorical variables were examined using the Chi-square test, and continuous variables underwent evaluation through either the Mann–Whitney U test or the Two-sample *t*-test. Statistical significance was declared at a two-sided *p*-value < 0.05. The intraclass correlation coefficient (ICC) was used to evaluate the reliability of DLCT 3D VOI-based quantitative parameters measurement. An ICC > 0.75 was considered good.

## Results

### Clinical features and DLCT 3D VOI-based quantitative parameters

In the cohort of 162 patients, 116 (71.6%) were classified into the low Ki-67 PI group, and 46 (28.4%) into the high Ki-67 PI group. The training set consisted of 83 patients (72.8%) with low Ki-67 PI and 31 patients (27.2%) with high Ki-67 PI, while the validation set included 33 patients (68.75%) with low Ki-67 PI and 15 patients (31.25%) with high Ki-67 PI. Table 1 presents the DLCT 3D VOI-based quantitative parameters and clinical features of PDAC in both the training and validation sets. Table 2 presents the DLCT 3D VOI-based quantitative parameters and clinical features in the high and low Ki-67 PI groups. Based on the results of univariate analysis, significant differences in clinical features, including age and CA125, were observed between the two groups (*p* < 0.05). The high Ki-67 PI group exhibited lower values for both 3D VOI-IC and 3D VOI-Zeff compared to the low Ki-67 PI group (*p* < 0.05). However, the other clinical and DLCT variables didn’t show statistically significant differences between the two groups (*p* > 0.05).

**Table 1:** Clinical and DLCT variables of PDAC patients

Variables	Training set (*n* = 114)	Validation set (*n* = 48)
Clinical features		
Age, years	62 (53, 68)	64 (55, 71)
BMI	22.03 (20.23, 24.17)	21.55 (20.46, 24.09)
PLR	209.93 ± 114.01	219.53 ± 101.97
NLR	4.29 ± 2.51	4.51 ± 3.12
LMR	3.30 ± 2.23	2.77 ± 1.53
PWR	37.71 ± 16.04	39.61 ± 19.97
Tbil	64.70 ± 92.68	75.25 ± 97.69
Albumin	39.90 (36.63, 41.90)	36.50 (34.45, 38.83)
Gender, *n* (%)		
Male	63 (55.3)	31 (64.6)
Female	51 (44.7)	17 (35.4)
CA19-9, *n* (%)		
Normal	19 (16.7)	12 (25.0)
Elevated	95 (83.3)	36 (75.0)
CEA, *n* (%)		
Normal	81 (71.1)	35 (72.9)
Elevated	33 (28.9)	13 (27.1)
CA125, *n* (%)		
Normal	62 (54.4)	32 (66.7)
Elevated	52 (45.6)	16 (33.3)
DLCT 3D VOI-based quantitative parameters		
3D VOI-IC	1.55 (1.24, 1.96)	1.69 (1.22, 1.87)
3D VOI-40keV (HU)	137.92 (101.77, 175.82)	134.08 (95.96, 186.02)
3D VOI-100keV (HU)	46.84 (41.82, 54.51)	47.92 (39.81, 54.60)
3D VOI-λHU	1.56 (1.00, 2.07)	1.44 (0.96, 2.22)
3D VOI-Zeff	8.07 (7.86, 8.36)	8.01 (7.75, 8.29)

*DLCT* dual-layer detector spectral computed tomography, *PDAC* pancreatic ductal adenocarcinoma, *BMI* body mass index, *PLR* platelet-to-lymphocyte ratio, *NLR* neutrophil-to-lymphocyte ratio, *LMR* lymphocyte-monocyte ratio, *PWR* platelet-to-white blood cell ratio, *Tbil* total bilirubin, *IC* iodine concentration, *λHU* slope of the spectral Hounsfield unit curve, *Zeff* effective atomic number, *3D* three-dimensional, *VOI* volume of interest, *CA125* carbohydrate antigen 125, *CA19-9* carbohydrate antigen 19-9, *CEA* carcinoembryonic antigen

**Table 2:** Univariate analysis to differentiate between high and low Ki-67 PI groups in the training set

Variables	High Ki-67 PI group (Ki-67 PI ≥ 50%, *n* = 31)	Low Ki-67 PI group (Ki-67 PI < 50%, *n* = 83)	F/Z/c²	*p*-value
Clinical features				
Age, years	66.00 (58.50, 69.50)	59.00 (51.00, 67.00)	−2.578	0.010
BMI	21.51 (19.99, 23.44)	22.06 (20.26, 24.41)	−1.035	0.301
PLR	224.59 ± 116.20	204.45 ± 112.69	0.006	0.417
NLR	4.35 ± 1.78	4.27 ± 2.73	4.295	0.879
LMR	2.86 ± 1.28	3.46 ± 2.47	1.210	0.202
PWR	37.83 ± 15.17	37.66 ± 16.35	0.056	0.959
Tbil	72.5 ± 96.15	61.79 ± 91.18	0.343	0.587
Albumin	39.90 (35.85, 42.05)	39.9 (36.95, 41.85)	−0.344	0.731
Gender, *n* (%)			0.135	0.713
Male	18 (58.1)	45 (54.2)		
Female	13 (41.9)	38 (45.8)		
CA19-9, *n* (%)			2.561	0.110
Normal	8 (25.8)	11 (13.3)		
Elevated	23 (74.2)	72 (86.7)		
CEA, *n* (%)			0.884	0.347
Normal	20 (64.5)	61 (73.5)		
Elevated	11 (35.5)	22 (26.5)		
CA125, *n* (%)			25.12	< 0.001
Normal	5 (16.1)	57 (68.7)		
Elevated	26 (83.9)	26 (31.3)		
DLCT 3D VOI-based quantitative parameters				
3D VOI-IC	1.15 (0.80, 1.32)	1.72 (1.43, 2.05)	−6.123	< 0.001
3D VOI-40keV (HU)	133.80 (105.47, 164.98)	139.69 (96.19, 181.09)	−0.487	0.626
3D VOI-100keV (HU)	46.32 (42.94, 54.14)	47.50 (40.73, 54.44)	−0.338	0.736
3D VOI-λHU	1.45 (1.02, 1.84)	1.57 (0.99, 2.16)	−0.681	0.496
3D VOI-Zeff	7.97 (7.78, 8.11)	8.15 (7.93, 8.44)	−2.771	0.006

*DLCT* dual-layer detector spectral computed tomography, *BMI* body mass index, *PLR* platelet-to-lymphocyte ratio, *NLR* neutrophil-to-lymphocyte ratio, *LMR* lymphocyte-monocyte ratio, *PWR* platelet-to-white blood cell ratio, *Tbil* total bilirubin, *IC* iodine concentration, *λHU* slope of the spectral Hounsfield unit curve, *Zeff* effective atomic number, *3D* three-dimensional, *VOI* volume of interest, *PI* proliferation index, *CA125* carbohydrate antigen 125, *CA19-9* carbohydrate antigen 19-9, *CEA* carcinoembryonic antigen

### Interobserver reproducibility

High interobserver agreement was observed for each DLCT 3D VOI-based quantitative parameter, with all ICC values of > 0.75. The detailed results are shown in Table S1.

### Development of predictive models for Ki-67 PI

Table 3 displays the multivariate logistic regression analysis for all significant variables (*p* < 0.05). CA125 (OR = 11.400; 95% CI: 3.936–33.022; *p* < 0.001) and 3D VOI-IC (OR = 0.014; 95% CI: 0.002–0.084; *p* < 0.001) were identified as significant through multivariate logistic regression. Subsequently, the clinical, DLCT, and DLCT-clinical models were developed using the identified independent risk factors. These models were independently validated in the validation set.

**Table 3:** Multivariate logistic regression analysis in the training set

Variables	Odds ratio	95% CI	*p*-value
CA125	11.400	3.936–33.022	< 0.001
3D VOI-IC	0.014	0.002–0.084	< 0.001

*CI* confidence interval, *IC* iodine concentration, *3D* three-dimensional, *VOI* volume of interest, *CA125* carbohydrate antigen 125

### Diagnostic effectiveness of the three models

**Fig. 2:**
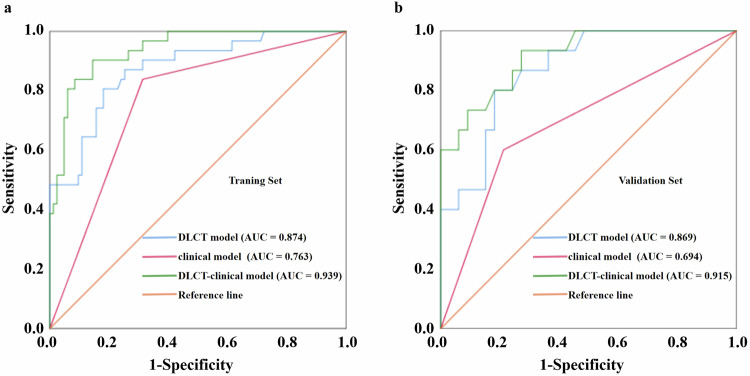
ROC depicting the predictive performance of the clinical, DLCT, and DLCT-clinical models for Ki-67 PI in PDAC (**a**, **b**). DLCT, dual-layer detector spectral computed tomography; AUC, area under the curve; PI, proliferation index; PDAC, pancreatic ductal adenocarcinoma; ROC, receiver operating characteristic

**Fig. 3:**
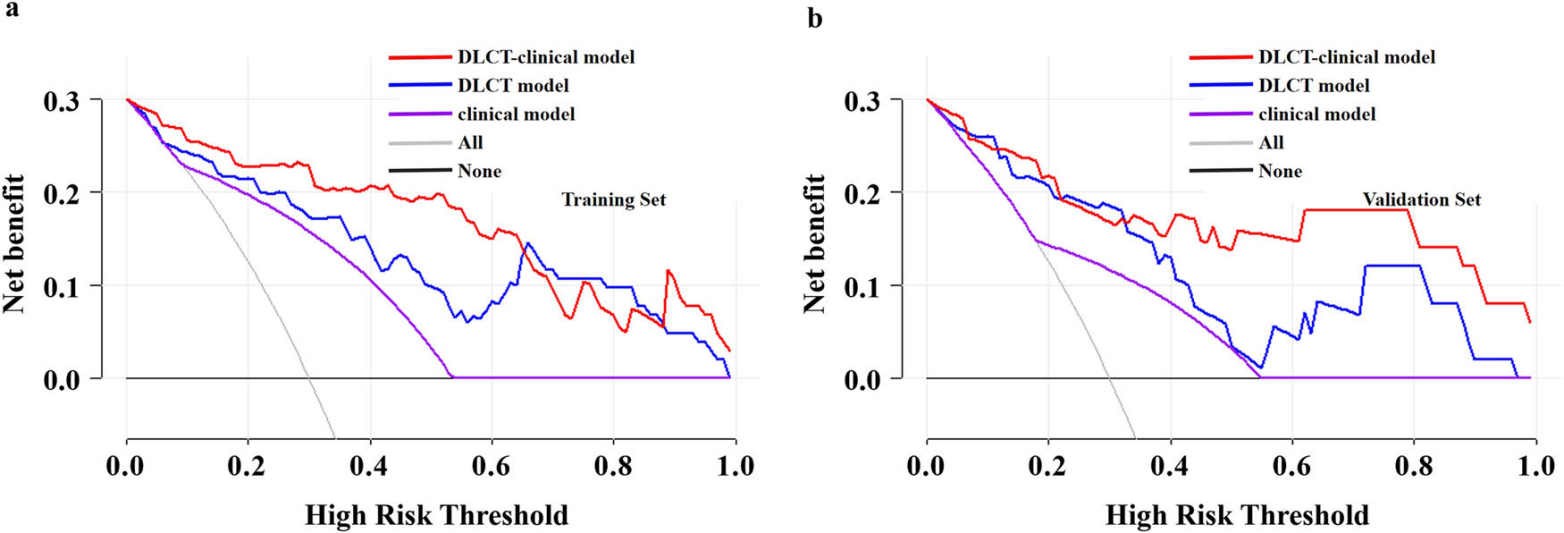
DCA results for the clinical, DLCT, and DLCT-clinical models (**a**, **b**). DCA, decision curve analysis; DLCT, dual-layer detector spectral computed tomography; PI, proliferation index

The diagnostic effectiveness of the three models is summarized in Table 4. ROC and DCA analyses conducted to distinguish high and low Ki-67 PI in both the training and validation sets for the three models are shown in Figs. 2 and 3. The clinical model demonstrated moderate diagnostic effectiveness in predicting Ki-67 PI among PDAC, achieving AUCs of 0.763 (95% CI: 0.666–0.859) in the training set and 0.694 (95% CI: 0.525–0.863) in the validation set. Both the DLCT model and the DLCT-clinical model exhibited superior diagnostic effectiveness in identifying Ki-67 PI in PDAC, yielding AUCs of 0.874 (95% CI: 0.801–0.946) and 0.939 (95% CI: 0.895–0.982) in the training set. In the validation set, the DLCT model and DLCT-clinical model achieved corresponding AUCs of 0.869 (95% CI: 0.768–0.969) and 0.915 (95% CI: 0.834–0.996). The DeLong test revealed a significant increase in the AUCs of the DLCT-clinical model compared to the clinical model in both the training (*p* < 0.001) and validation sets (*p* = 0.001). However, no significant differences in performance were observed between the DLCT model and the clinical model in both the training (*p* = 0.070) and validation sets (*p* = 0.076). DCA indicated that the DLCT-clinical model yielded a greater net benefit across threshold probabilities ranging from 0.04 to 0.96 in the training set and 0.04 to 0.95 in the validation set.

**Table 4:** Diagnostic effectiveness of the clinical, DLCT, and DLCT-clinical models

Models	Training set				Validation set			
	AUC (95% CI)	SEN	SPE	DeLong	AUC (95% CI)	SEN	SPE	DeLong
Clinical model	0.763 (0.666–0.859)	0.839	0.687	0.070^a^	0.694 (0.525–0.863)	0.600	0.788	0.076^a^
DLCT model	0.874 (0.801–0.946)	0.806	0.819	0.012^b^	0.869 (0.768–0.969)	0.800	0.818	0.261^b^
DLCT-clinical model	0.939 (0.895–0.982)	0.903	0.855	< 0.001^c^	0.915 (0.834–0.996)	0.933	0.727	0.001^c^

*AUC* area under the receiver operating characteristic curve, *CI* confidence interval, *DLCT* dual-layer detector spectral computed tomography, *SEN* sensitivity, *SPE* specificity

^a^ Clinical model versus DLCT model

^b^ DLCT model versus DLCT-clinical model

^c^ DLCT-clinical versus clinical model

### Development and performance for DLCT-clinical nomogram

**Fig. 4:**
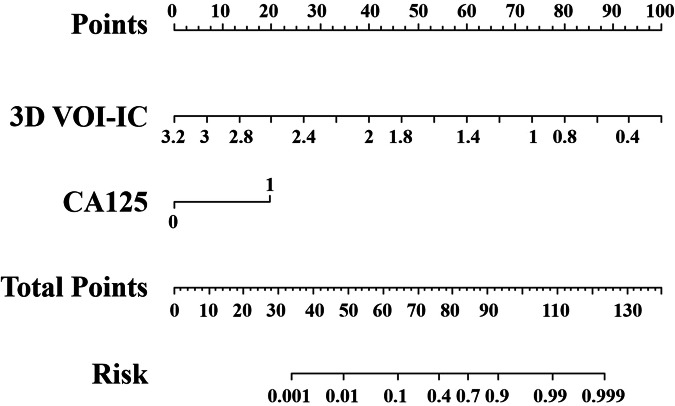
DLCT-clinical nomogram was developed in the training set, incorporating the 3D VOI-IC and CA125. DLCT, dual-layer detector spectral computed tomography; IC, iodine concentration; CA125, carbohydrate antigen 125; 3D, three-dimensional; VOI, volume of interest

**Fig. 5:**
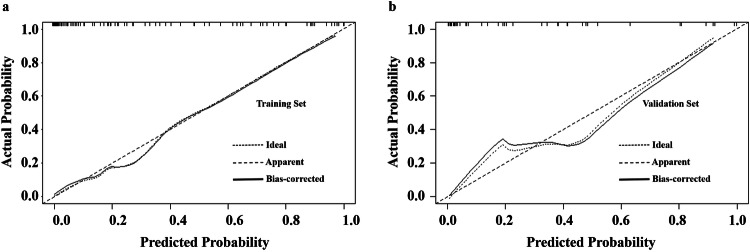
Calibration curves of DLCT-clinical nomogram (**a**, **b**). DLCT, dual-layer detector spectral computed tomography

**Fig. 6:**
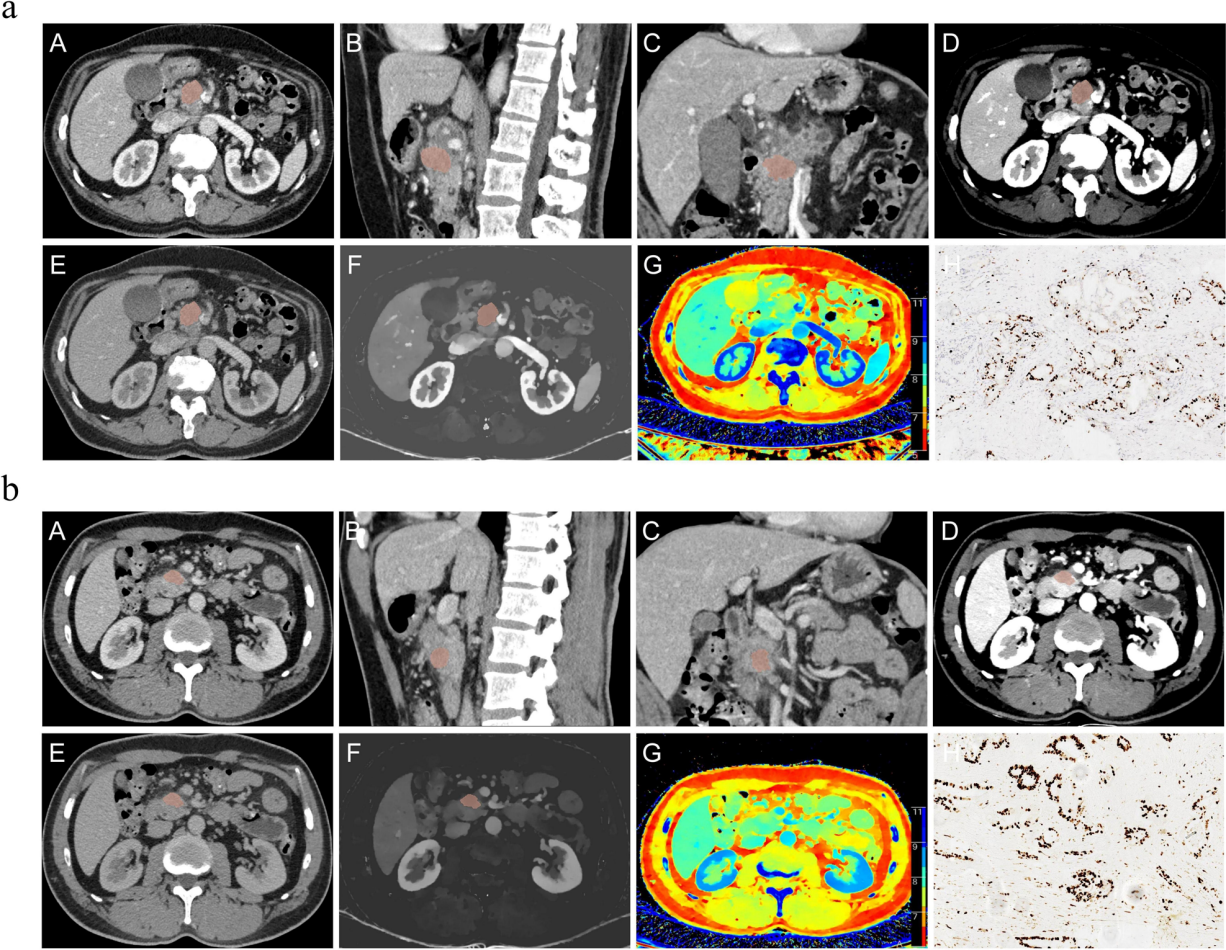
**a** A 64-year-old male with low Ki-67 PI in PDAC. **A**–**C** Conventional PVP imaging reveals a 3D-VOI. **D** The 40 keV PVP image shows a 3D-VOI CT value of 144.45 HU. **E** The 100 keV PVP image shows a 3D-VOI CT value of 46.5 HU. **F** The PVP iodine map reveals a 3D-VOI IC value of 1.32 mg/mL. **G** The PVP effective atomic number map shows 3D-VOI Zeff value of 8.05. **H** Ki-67 IHC staining demonstrates that approximately 30% of cells are positive for nuclear staining. **b** A 50-year-old male with high Ki-67 PI in PDAC. **A**–**C** Conventional PVP imaging reveals a 3D-VOI. **D** The 40 keV PVP image shows a 3D-VOI CT value of 129.26 HU. **E** The 100 keV PVP image shows a 3D-VOI CT value of 46.98 HU. **F** The PVP iodine map reveals a 3D-VOI IC value of 1.1 mg/mL. **G** The PVP effective atomic number map shows 3D-VOI Zeff value of 7.94. **H** Ki-67 IHC staining demonstrates that approximately 70% of cells are positive for nuclear staining

The DLCT-clinical nomogram was formulated by integrating the selected DLCT 3D VOI-based quantitative parameters and clinical features (Fig. 4), each multiplied by their corresponding scaled coefficients. The equation was expressed as follows: DLCT-clinical nomogram = −5.113*3D VOI-IC + 3.023*CA125 + 4.289. The calibration curve analysis demonstrated a satisfactory consistency between the predicted and actual probabilities of the nomogram in predicting Ki-67 PI in PDAC for both the training and validation sets (Fig. 5). Representative images are shown in Fig. 6.

## Discussion

In this retrospective study, DLCT 3D VOI-based quantitative parameters and clinical features were utilized to evaluate the Ki-67 PI in PDAC. The findings revealed that both 3D VOI-IC and CA125 emerged as independent risk factors in the analysis, and PDAC with high Ki-67 PI group displayed abnormal CA125 levels and higher 3D VOI-IC value than those with low Ki-67 PI group. Based on these findings, we developed and validated a DLCT-clinical model for non-invasively predicting Ki-67 PI in PDAC preoperatively. In predicting Ki-67 PI, the DLCT-clinical model showed superior identification performance compared to both the clinical and DLCT models in both the training and validation sets. These findings indicate that the DLCT-clinical model could function as a robust imaging tool, assisting clinicians in the preoperative and non-invasive screening of PDAC patients with a high Ki-67 PI.

Iodine image depicts the iodine content distribution in various tissues, and the 3D VOI-IC values quantified on these images contribute to reflecting the degree of tumor neovascularization and iodine deposition in the tissue [20]. Research has shown that the contrast media fails to fill the microvessels completely and quickly in the arterial phase [21]. However, compared to the arterial phase, PVP exhibits a prolonged delay following contrast injection, allowing sufficient time for contrast to fill the microvasculature and permeate the basement membrane into the cellular interstitium [22]. Hence, the 3D VOI-IC in PVP provides a more effective representation of tumor blood flow distribution. Furthermore, our analysis exclusively focused on this phase because certain studies have shown significant potential in PVP images for providing information on the biology of PDAC [23, 24].

In this study, we noticed a substantial decrease in 3D VOI-IC in the high Ki-67 PI group compared to the low Ki-67 PI group in the training set (1.15 vs. 1.72, *p* < 0.001), which might be attributed to the active cell proliferation associated with high Ki-67 PI group. In PDAC, heightened cell proliferation leads to ischemia and hypoxia, establishing a microenvironment conducive to PDAC growth [25]. Moreover, PDAC is characterized by lower vascularity and a substantial fibrous stromal component [26]. PDAC patients with a high Ki-67 PI exhibit reduced iodine accumulation and lower IC values when intravenous contrast media is administered, making it challenging for the iodine contrast agent to penetrate the tumor parenchyma. Hence, the IC could serve as one of the parameters for predicting Ki-67 PI in PDAC. Nevertheless, it is crucial to recognize the influence of individual variability on IC [20]. To more accurately assess IC and its relationship to Ki-67 PI, we employed 3D quantitative analysis. Compared to traditional two-dimensional (2D) methods, 3D analysis provides more precise and reproducible results, reducing IC variability and enhancing the prediction of clinical outcomes [27]. Unlike 2D analysis, which may only capture part of the tumor, 3D analysis includes the entire tumor, offering a more comprehensive assessment and minimizing observer bias. Therefore, studies utilizing 3D analysis are more likely to yield accurate and reliable results compared to those using conventional 2D methods [28, 29].

Our study also found that the clinical feature CA125 could distinguish Ki-67 PI in PDAC. The high Ki-67 PI group demonstrated a markedly elevated positive rate of CA125 in comparison to the low expression group (76.1% vs. 28.4%). The clinical model developed using this variable showed moderate diagnostic effectiveness, achieving an AUC of 0.763 in the training set and 0.694 in the validation set. Elevated CA125 levels serve as a crucial indicator of a high tumor burden and a propensity for metastasis in PDAC, potentially indicating an unfavorable prognosis for PDAC [30]. Previous research has indicated that CA125 levels exhibit the strongest association with the presence of occult metastasis compared to other tumor markers like CA19-9 and CEA [31]. Ki-67 PI reflects the degree of tumor proliferation activity, with a higher expression indicating more active tumor proliferation, correlating with an increased risk of metastasis and an unfavorable prognosis [5, 6]. Consequently, the high Ki-67 PI group demonstrated a greater inclination toward elevated CA125 levels.

Further, a DLCT-clinical nomogram derived from the DLCT-clinical model integrating 3D VOI-IC and CA125 was developed, which demonstrated the highest diagnostic effectiveness in predicting Ki-67 PI and achieving AUCs of 0.939 and 0.915 in the training and validation sets, respectively. The AUCs exhibited a significant enhancement between the DLCT-clinical model and the clinical model in both the training set (*p* < 0.001) and the validation set (*p* = 0.001). The DCA demonstrated that patients derived a greater net benefit from the DLCT-clinical nomogram compared to the DLCT and clinical models in the two sets, underscoring the clinical applicability of the DLCT-clinical nomogram. In addition, the calibration curves displayed a satisfactory consistency between the nomogram predictions and the actual observed probability. The results suggest that the DLCT model and clinical model complement each other, and their integration could improve the accuracy of predicting Ki-67 PI in PDAC.

This study had several limitations. Firstly, the retrospective study may have introduced a potential for selection bias. Secondly, the DLCT-clinical model underwent validation using data from a single institution, which might impact the generalizability of the results. External validation of the DLCT-clinical model was deemed necessary. Currently, we are actively advancing this work as well, which may be realized in the future as the sample size expands. Thirdly, some patients had biopsy-confirmed PDAC and underwent Ki-67 PI testing but were not eligible for radical resection. This may introduce sampling bias, as biopsy specimens might not adequately capture tumor heterogeneity. However, many patients presented with unresectable locally advanced or metastatic disease at diagnosis and required needle biopsy for confirmatory diagnosis and treatment planning. This situation may be more applicable to clinical practice.

## Conclusion

In conclusion, this study employed DLCT 3D VOI-based quantitative parameters and clinical features to develop a non-invasive and effective DLCT-clinical model that demonstrates favorable predictive performance for preoperatively predicting Ki-67 PI in PDAC patients. The visualized nomogram may assist clinicians in identifying PDAC patients with a high Ki-67 PI and realizing individualized management of PDAC patients.

## Supplementary information

ELECTRONIC SUPPLEMENTARY MATERIAL

## Data Availability

The datasets used and/or analyzed during the current study are available from the corresponding author upon reasonable request.

## Abbreviations

AUC: Area under the receiver operating characteristic curve
CA125: Carbohydrate antigen 125
CA19-9: Carbohydrate antigen 19-9
CEA: Carcinoembryonic antigen
CI: Confidence interval
DCA: Decision curve analysis
DLCT: Dual-layer detector spectral computed tomography
IC: Iodine concentration
IHC: Immunohistochemical
PDAC: Pancreatic ductal adenocarcinoma
PI: Proliferation index
PVP: Portal venous phase
ROC: Receiver operating characteristic curve
ROI: Region of interest
VOI: Volume of interest
Zeff: Effective atomic number
λHU: Slope of the spectral attenuation curves
2D: Two-dimensional
3D: Three-dimensional
