# Supplementary material for: Development and validation of a model based on preoperative dual-layer detector spectral computed tomography 3D VOI-based quantitative parameters to predict high Ki-67 proliferation index in pancreatic ductal adenocarcinoma
Source: Insights Imaging. 2024 Dec 5;15:291. doi: 10.1186/s13244-024-01864-9 (PMC11621245; doi:10.1186/s13244-024-01864-9)
Supplement: Supplementary file 1 — ELECTRONIC SUPPLEMENTARY MATERIAL [file 13244_2024_1864_MOESM1_ESM.pdf]

Development and validation of a model based on preoperative dual-layer detector spectral computed tomography 3D VOI-based quantitative parameters to predict high Ki-67 proliferation index in pancreatic ductal adenocarcinoma

ELECTRONIC SUPPLEMENTARY MATERIAL

Table S1 Interobserver agreement of DLCT 3D VOI-based quantitative parameters measurement of tumor

Parameters	R1(n = 162)	R2(n = 162)	ICC(95%CI)
3D VOI-IC	1.59(1.23,1.94)	1.57(1.14,1.97)	0.874(0.832,0.906)
3D VOI-40keV (HU)	136.58(97.63,179.57)	134.72(98.11,179.46)	0.878(0.837,0.909)
3D VOI-100keV (HU)	47.33(40.66,54.43)	47.23(38.04,54.31)	0.903(0.859,0.932)
3D VOI-λHU	1.54(0.98,2.16)	1.50(1.00,2.15)	0.856(0.808,0.892)
3D VOI-Zeff	8.02(7.75,8.26)	8.01(7.68,8.37)	0.898(0.863,0.924)

Parameters are expressed as median (25th, 75th percentiles). DLCT, dual-layer detector spectral computed tomography; 3D, three-dimensional; VOI, volume of interest; IC, iodine concentration; λHU, slope of the spectral Hounsfield unit curve; Zeff, effective atomic number; ICC, intraclass correlation coefficient; CI, confidence interval; R1, radiologist 1; R2, radiologist 2.
